# A Treatise on the Nature, Cause, and Treatment of Erysipelas

**Published:** 1844-06

**Authors:** 


### REVIEWS.

---

ART. III.—*A Treatise on the Nature, Causes, and Treatment of Erysipelas.* By THOMAS NUNNELEY, Lecturer on Anatomy, Physiology, and Pathology, in the Leeds School of Medicine; Surgeon to the general Eye and Ear Infirmary; Honorary Secretary to the Philosophical and Literary Society, &c. &c. Philadelphia: Ed. Barrington & Geo. D. Haswell. 1vol. 12mo. pp. 235. 1844.

Our profession is under many obligations to Dr. Bell, of Philadelphia, whose judgment, independence and learning fit him very eminently for the editorial tripod, which he has occupied for many years. As a critic his decisions are entitled to high respect, and the works which have been re-published by his directions have been stamped by so much excellence, that it is a sufficient passport to public favor that a volume has appeared in the *Select Medical Library.* The work which stands at the head of this article has been published very opportunely. At a time when erysipelas is prevalent in many parts of the country, and in some has assumed a highly malignant form, a treatise describing all its varieties, and the best modes of treatment—in a word, a full and complete monograph on the subject—will be highly acceptable to the profession. Dr. Bell has judiciously added to the work of Nunneley an account of epidemic erysipelas, as this disease has exhibited itself in this country. This portion of the volume will be read with deep interest by all, who have not made themselves acquainted with the nature of the disease which has prevailed with great mortality, in certain localities, under the name of "*black tongue.*" But the work in its entireness is one which will be accounted necessary to every physician's library. It is not our purpose to enter upon an extended review of it, but simply to notice such parts as it is supposed are especially interesting at this time.

Our author thinks the following affections may be grouped together, namely: erythema, eyrsipelas, diffuse inflammation, of the cellular membrane, puerperal fever, diffuse inflammation of the serous membranes, diffuse inflammation of the mucous membranes, arachnitis, diffuse phlebitis, and inflammation of the absorbents, all of which are treated of, to some extent, in this valuable work. The details under the various heads are curious and instructive, and those which relate to puerperal fever will be found exceedingly interesting. Among the inferences to be drawn from them is an important one, that the most serious consequences are apt to flow from a wound received on the hand or fingers in dissecting the body of a patient who has died of that complaint. Many cases are cited in which the pricking of the finger, in dissections of bodies affected with erysipelas, was followed by fatal disease. Most readers will be surprised to find puerperal fever and wounds of the dissecting room in the same class, and to see these grouped with the other forms of disease recited, but taking the facts stated by our author as true, we cannot see how his conclusions are to be resisted. We shall, however, pass from this question to matters of more practical moment.

Dr. Sutton's paper on epidemic erysipelas introduced into the volume by Dr. Bell, illustrates the extension of the disease to the mucous membranes. We quote the following from Dr. S.'s description of the complaint:

"This epidemic has assumed a variety of characters, often presenting a difference in different neighborhoods through which it passed. I have had an opportunity of becoming acquainted with the disease over a large section of country, having the most of the Wilmington practice to attend to, owing to the physician of that place having a severe attack of illness, during the time the disease was raging with the most violence; and from the intimate connection which appears to exist between the different characters of this disease, I have considered the epidemic as an inflammatory disease, of a peculiar character. It attacks the mucous membrane of the respiratory passages; the tongue; the glands of the throat; the skin in the form of erysipelas; the lungs and thoracic viscera; the uterus, and its appendages, producing puerperal fever; as this last disease in several places, has also accompanied the epidemic. This disease, in every variety, has had a tendency to assume a typhoid grade of fever, after it had continued a few days.

"The following is a synopsis of the simptoms of this epidemic. When the throat was the part attacked, after the usual premonitory symptoms, which have been frequently mentioned, had continued for two or three days, the patient was generally seized with a chill, which lasted, in many cases, four or five hours; this was followed by a high fever, swelling of the tonsils, submaxillary, parotid, and lymphatic glands of the neck; neuralgic pains, darting over the side of the neck and head, frequently following the temporal artery; tongue, covered at first with a thick brown coat, soon became swollen and often very dark in the centre; deglutition frequently very difficult; pulse generally full, though easily compressed; skin at first hot and dry, becoming moist and continuing so after venesection. In the mild form of the disease these symptoms were frequently removed at once by an active antiphlogistic course of treatment. Sometimes the mild form had only the appearance of cynanche tonsillaris. But in the more malignant form, where the throat was affected, after the above symptoms had continued for two or three days, and sometimes from the very commencement, the pharynx became of a dark purple color; this color generally spread over the palate, tongue, and sides of the cheeks, the tongue becoming very much swollen, assuming a blackish-brown color; deglutition in many cases was almost impossible. In most of these cases an erysipelas would commence at the angle of the mouth, or nose, and spread over the face and head, with all the symptoms peculiar to that disease. The inflammation of the throat was seldom stationary; sometimes passing down the trachea, with symptoms resembling laryngitis, or cynanche trachealis, and at last assuming the symptoms of pneumonia. Sometimes this inflammation passed into the nostrils, and from them into the frontal sinuses; sometimes apparently into the antrum maxillary, but in nearly every case that I saw, the *throat became well, while the erysipeles was spreading over the skin.*

"Sometimes this disease appeared to commence in the fron tal sinuses and antrum; large quantities of water would be dischared from the nose, a violent pain felt over the eyebrows, or one of the malar bones, the face becoming very much swollen, the swelling closing the eyelids. These symptoms generally continued until an erysipelas made its appearance, or there was a copious discharge of bloody mucus from the nose. In the case that I met with, the neck was enormously swollen, from the left ear down to the sternum, without any redness of the skin, or but little inflammation of the pharynx; this swelling rapidly subsided, and was followed by a profound coma that terminated in death. This disease seldom presented the putrid symptoms of *cynanche maligna* and in those cases that it did, I believe the cause might be traced to the imprudent use of mercury. In a number of cases that I met with, the inguinal glands were the seat of the disease, becoming very much inflamed, and an erysipelas first making its appearance there, and spreading over the abdomen."

*Treatment.*—Dr. Sutton used blood-letting from a large orifice, and the antiphlogistic treatment generally, bearing in mind the tendency of the disease to a typhoid character. Copious v. s. from a small orifice he thinks was injurious. He adds:

"When the throat was attacked, emetics, followed by mercurial cathartics, nauseants, blisters, liniments, and sinapisms to the throat, pediluvium, acidulated and pepper gargles, scarifying the tonslis, and when the throat was ulcerated, the application of a solution of nitrate of silver was the course generally adopted, and in a large number of cases the bleeding, the emetic, and mercurial cathartic cut short the disease at once. In administering mercury in this form of the disease, a few doses generally filled the indication, and, as I before mentioned, great caution was necessary; for wherever it produced its specifiic effect upon the mouth and salivary glands, I believe it was almost invariably attended by injurious consequences. When the erysipelas made its apperance upon the skin, it was treated according to the character that it assumed, and its accompanying fever. Alterative doses of calomel and ipecac, (carefully avoiding ptyalism), followed by saline cathartics, antimonial diaphoretics in the robust; wine whey, carbonate of ammonia, Dover's powder, in combination with calomel, followed by gentle laxatives, when the disease had assumed a typhoid character. As a local application to the erysipelas, a solution of the sulphate of copper, and the sulphate of iron as has been highly recommended, appeared to produce good effects. However, in many cases, when the skin was not blistered, influenced by the resemblance the disease had to a burn, I was induced to try the spirits of turpentine, which I thought produced the very best effects."

In the stage of collapse, quinine is to be given, in conjunction with the diffusible stimuli. Opium and tartrate of antimony are prescribed in conditions where the brain is affected, attended with a low muttering delirium.

Our author discusses the treatment of erysipilas at great length. The authorities for and against the various plans of cure recommended in the disease are fully stated, by which the reader will be able to gather the views of all the schools on the subject. Some insist upon blood-letting to the exclusion of stimulants, and others upon stimulants to the exclusion of venesection, while a third class, without forbidding the use of the lancet, recommend the use of stimulants. Emetics are in the same predicament—extolled by some, and condemned by others. Our author inclines to their employment. Even purgatives have not been able to command the unanimous suffrage of the profession; but Dr. N. justly regards them as amongst the most valuable remedies we possess in the treatment of erysipelas. Preparations of mercury, diaphoretics, and diuretics, digitalis, colchicum, antimony, narcotics, turpentine, and camphor, are enumerated as among the general means of cure, to be prescribed according to the judgment of the practitioner. The local remedies recommended, are bleeding, by cups, leeches, punctures, and incisions, cold lotions, stimulants, farinaceous powders, warm fomentations, ointments, the actual cautery, and blisters, nitrate of silver, tincture of iodine, and bandages. The treatment applicable in the different varieties of the disease is given at the close of the work, and will be found sufficiently detailed. We must refer the reader to the treatise itself for particulars, which would extend our notice beyond the limits necessarily assigned to it.

Erysipilas is a frequent disease of hospitals. The atmosphere of such institutions becomes tainted at times, and patients introduced with other diseases are attacked, from slight causes, with erysipellatous inflammation. During the few last months we have been presented with some striking illustrations of this fact in the Louisville Marine Hospital, where erysipelas has been showing itself in the subjects of other complaints. Several cases appeared in February, and others have existed at different times since, the disease having been induced by trivial injuries of the skin. In one instance it followed the application of a blister to a syphilitic node on the shin, but was easily arrested by drawing a line around the inflamed spot with nitrate of silver. In a second case, it succeeded to a slight surgical operation on a young man, and in that case was attended by alarming constitutional symptoms, which were only subdued by the most energetic treatment. In a third, it supervened upon leech-bites, and terminated fatally. The subject was a young man, eighteen years of age, who was taken to the hospital on account of insanity. He was of a delicate constitution, which had suffered much from indigestion. Leeching having been practiced over the epigastrium, and again, in two days, on the spine, between the shoulders, erysipilas made its appearance at both places, and from the latter spread over the neck, and a portion of the scalp, extending to the cheek, and downwards half way the back, and proved fatal in eight days after it exhibited itself. The depletion by the leeches and purgatives, with the use of extract of hyosciamus and stramonium, had restored the patient to a sound mind before the erysipelas broke out. Delirium supervened upon the extension of the inflammation over the neck, and persisted as long as the young man lived. The nitrate of silver seemed to arrest the disease on the abdomen, when it first broke out, but did not appear to exert much influence upon it as it advanced along the neck towards the head. This caustic is the favorite remedy of our practitioners for limiting the disease, which it is found to do in many cases. The corrosive sublimate we have also seen applied with decided advantage. With both, the object is to vesicate the skin in advance of the inflammation. The constitutional remedies have already been recited, and the disease being one of the constitution our reliance is properly upon them. Y.

